# The Effects of *In Vitro* Maturation Technique on The Expression of Genes Involved in Embryonic Genome Activation of Human Embryos

**DOI:** 10.22074/cellj.2018.4804

**Published:** 2018-01-01

**Authors:** Parvin Dorfeshan, Marefat Ghaffari Novin, Mohammad Salehi, Reza Masteri Farahani, Fatemeh Fadaei-Fathabadi, Ronak Sehatti

**Affiliations:** 1 Department of Biology and Anatomical Sciences, Faculty of Medicine, Shahid Beheshti University of Medical Sciences, Tehran, Iran; 2 Infertility and Reproductive Health Research Center, Shahid Beheshti University of Medical Sciences, Tehran, Iran; 3 Cellular and Molecular Biology Research Center, Shahid Beheshti University of Medical Sciences, Tehran, Iran; 4 Department of Biotechnology, School of Advanced Technologies in Medicine, Shahid Beheshti University of Medical Sciences, Tehran, Iran; 5 Infertility and Reproductive Health Research Center, Aban Hospital, Tehran, Iran

**Keywords:** Embryonic Development, Intracytoplasmic Sperm Injection, *In Vitro* Maturation, Ovarian Stimulation

## Abstract

**Objective:**

*In vitro* maturation technique (IVM) is shown to have an effect on full maturation of immature oocytes and the subsequent embryo development. Embryonic genome activation (EGA) is considered as a crucial and the first process after fertilization. EGA failure leads to embryo arrest and possible implantation failure. This study aimed to determine the role of IVM in EGA-related genes expression in human embryo originated from immature oocytes and recovered from women receiving gonadotrophin treatment for assisted reproduction.

**Materials and Methods:**

In this experimental study, germinal vesicle (GV) oocytes were cultured *in vitro*. After intracytoplasmic sperm injection of the oocytes, fertilization, cleavage and embryo quality score were assessed in vitro and *in vivo*. After 3-4 days, a single blastomere was biopsied from the embryos and then frozen. Afterwards, the expression of EGA-related genes in embryos was assayed using quantitative reverse transcriptase-polymerase chain reaction (PCR).

**Results:**

The *in vitro* study showed reduced quality of embryos. No significant difference was found between embryo quality scores for the two groups (P=0.754). The *in vitro* group exhibited a relatively reduced expression of the EGA- related genes, when compared to the *in vivo* group (all of them showed P=0.0001).

**Conclusion:**

Although displaying the normal morphology, the IVM process appeared to have a negative influence on developmental gene expression levels of human preimplanted embryos. Based on our results, the embryo normal morphology cannot be considered as an ideal scale for the successful growth of embryo at implantation and downstream processes.

## Introduction

Subsequent to *in vitro* maturation (IVM), as a novel expanded technique, improved immature oocyte can be used to treat infertile women. A variety of studies showed successful fertilization, development of embryo and pregnancies with immature human oocytes matured *in vitro* (1, 2). However, other studies in this field revealed that the blastocyst performance of IVM is limited (3, 4). 

Before implantation stage, embryo undergoes various processes including oocyte maturation, activation of embryonic genome and a transcription shift from maternal to embryonic controls (5). Therefore, IVM process could be effective on the oocytes quality as any growth rate intervention would influence maturation of oocyte and further embryo development (6).

During female period, maternal proteins/transcripts control the embryonic developmental program until occurrence of embryonic genome activation (EGA). EGA is a process that occurs upon the first maternal mRNA degradation. After this stage, EGA occurrence would happen at the human embryos in 4to 8-cell stage (7, 8). In one study, the gene expression profile comparison in steps 5to 8-cell embryos, considering the single blastomere level, was performed and revealed novel EGA-related genes including *CCT3, DPPA5, MYC, POU5F1* and *CDH1*. One of the crucial stages of embryo development in 5to 8-cell stage is EGA, while the failure of this stage leads to embryo arrest and final implantation failure (9).

Generally, morphological features of oocyte and embryo are common criteria for determining embryonic developmental competence to select the greatest viability (10, 11). A successful pregnancy establishment cannot rely on normal morphology of embryo. The comparison of bovine morulae and blastocysts between culture systems and protein supplements has revealed variations in the comparative increase of some gene transcripts which are developmentally important (12, 13). 

Accordingly, these gene expressions could be a potentially substantial marker for evaluating embryo viability and implantation (3). Several researches revealed that there are significant variations in mRNA levels of some genes produced *in vitro* and *in vivo* embryos (14-17). Currently, determination of mRNA abundance in bovine during primary stage of embryonic development serves as an appropriate quality marker for both *in vitro* and culture conditions techniques (14, 18, 19). However, in the assisted reproductive treatment (ART) for clinical practice, it would be difficult to apply these important markers at the present time. Major research is needed for the development of reliable markers to evaluate oocyte quality and embryo viability (3).

Given the above, this study attempted to identify representative genes to characterize the EGA-related genes in human embryos. Moreover, this research is going to answer whether these gene expressions are influenced by IVM culture during the growth phase of oocytes. We aimed to provide the first library of EGA-related gene expressions within human embryos, resulting from intracytoplasmic sperm injection into oocytes matured *in vitro* compared to oocytes matured *in vivo*, to lay the foundations for subsequent studies. 

## Materials and Methods

This project was approved by the Ethics Committee of Shahid Beheshti University of Medical Sciences, Tehran, Iran (SBMU.REC.1393.78). In this research, written and verbal informed consent was obtained from the couples who were undergone ICSI/preimplantation genetic diagnosis (PGD) conforming to standard protocols. The research conducted from April 2014 to June 2016, including 19 couples who performed ICSI-PGD cycles and 45 couples who underwent repeated implantation failure (RIF) treatment. Two different experiments were studied. We used immature oocytes at the GV stage obtained from patients who underwent RIF treatment (*in vitro* group). In addition, we used normal biopsied embryos excess donated for doing scientific research after embryo transfer (*in vivo* group). Considering the aim of study, data relevant to maturation rate of immature oocytes at the GV stage was evaluated through *in vitro* group. Fertilization rate (16-19 hours after sperm injection), cleavage rate and quality score of embryos (72 hours after sperm injection) were examined in the two groups. 

In this study, female factor infertility, including chronic anovulation, poly cystic ovarian syndrome (PCOS), endometriosis, low ovarian reserve (defined as five or fewer oocytes on retrieval) and male factor infertility were the exclusion criteria. Female and male patients were less than 35 and 45 years old, respectively. 

## Controlled ovarian stimulation

A long protocol regimen was applied to control ovarian stimulation, consisting of gonadotrophin releasing hormone (GnRH) agonist. Patients’ hypophyseal suppression was applied by 0.1 mg administration of Decapeptyl (triptorelin, Ferring Pharmaceutical, Germany) or Diphereline (triptorelin, Ipsen Pharma, France) in the previous cycle midluteal phase. The GnRH analogue dose was decreased to 0.05 mg until the day of administering human chorionic gonadotrophin (hCG).

For ovarian stimulation of the patients, recombinant follicle-stimulating hormone (FSH, Gonal-F, MerckSerono, Germany), human menopausal gonadotrophin (hMG, Menopur, Ferring Pharmaceuticals) or high purified urinary FSH (Fostimon, IBSA, Switzerland) was used. Transvaginal ultrasound was used on the day 5 of stimulation to evaluate the effect of hormone therapy on ovary. Subsequently, gonadotrophin doses were individualized based on patients’ results. While two or more follicles reached a mean diameter of 18-20 mm, ovulation was triggered with 10,000-IU HCG (IBSA). After 34-36 hours of hCG injection, oocyte retrieval was carried out by transvaginal ultrasound guided follicle aspiration.

## Oocyte insemination

To digest the mass of cumulus-oocyte complexes, hyaluronidase enzyme (Life Global, USA) was used after oocyte retrieval. Metaphase II (MII) oocytes with first polar body were used for intracytoplasmic sperm injection (ICSI) (*in vivo* group). In couples with RIF treatment, GV oocytes (*in vitro* group) were collected. The oocytes were incubated in 6% CO_2_ and 37°C (Memmert, Germany) under mineral oil (Irvine Scientific, USA) for approximately one hour. They were subsequently denuded with 1% hyaluronidase enzyme (Life Global, USA) and a hand-drawn glass pipette. 

For GV oocytes detection (*in vitro* group), an invert microscope (SM2800, Nikon) with 200 fold magnification was used. Our strategy for selecting GV oocytes was prominent nucleus in the cytoplasm homogeneous and the oocytes without any defects in the overall appearance. The GV oocytes were placed in a 20-30 µl drop of a commercial IVM medium (oocyte maturation medium, Sage Media, USA) supplemented with 75 IU/ml FSH and 75 IU/ml luteinizing hormone (LH) according to the manufacturer’s instructions for 24-30 hours. Oocytes with the first polar body could subsequently be injected by ICSI. 

## Sperm preparation 

After 2-3 days of abstinence, semen samples were collected via masturbation into a non-toxic sterile container. After the liquefaction of the semen at 37°C, 5% CO_2_ in air for 30 minutes, we processed the samples by swim-up technique.

One milliliter of semen sample was placed in a sterile conical centrifuge tube and a 1.2 ml layer of the medium (Ham’s F-10, Sigma, USA) was poured gently over it. The sample was centrifuged at 300-500 g for 5 minutes and the supernatant was discarded. The pellet was diluted with 0.5 ml of medium. The tube was inclined at an angle of about 45° and was incubated for 1 hour at 37°C. After the swim-up technique, two aliquots (200 ml) of the processed spermatozoa were taken, one of which was used for sperm parameters assay and the other was used for ICSI. According to World Health Organization standard, sperm parameters, including sperm concentration, morphology and motility were determined as more than 15×10^6^ spermatozoa per ml, more than 4% and more than 32%, respectively (20). 

## Evaluation of fertilization, cleavage rate and embryo quality score

After injection, oocytes were incubated in 20 µl droplets of global total medium (Life Global) under equilibrated mineral oil (in a humidified atmosphere, 5% CO_2_ at 37°C). After 16-18 hours, the oocytes were tested for pronuclei appearance (conforming to the conventional routine practice). Zygotes were cultured in global total medium (Life Global) and on day 3, their developmental stage was evaluated.

After 16-19 hours of sperm injection, fertilization rate was determined as the resulting zygotes percentage by counting quantity of two pronucleus cells from the injected MII oocytes total number. Embryos were scored for their quality 72 hours after ICSI. Morphology of the embryos was calculated according to previous research in this area (21). To determine the rate of cleavage, the cleaved fertilized embryos total number was computed on day 3 (22).

## Blastomere biopsy 

On day 3, post-ICSI, embryos were inserted in each microdrop of 5 µl of Ca^2+^/Mg^2+^ free biopsy medium (LG PGD BIOPSY medium, Life Global) under mineral oil. After mechanical drilling of the zona pellucida, one blastomere was gently removed by a biopsy micropipette for analysis. The embryo was washed two times in global total medium (Life Global) and transferred into a fresh medium drop for embryo vitrification. For 15 minutes at room temperature (2027.), the embryo was maintained in an equilibration solution (Kitazato BioPharma Co., Japan). Afterwards, the embryos were aspirated for 1 minute period and then inserted into the vitrification solution (VS, Kitazato BioPharma Co.) at room temperature. Subsequently, the embryos were inserted on cryotop with a minimum volume of VS solution, and quickly plunged into liquid nitrogen (23). 

## RNA isolation, cDNA synthesis and quantitative reverse transcriptase-polymerase chain reaction 

In this research, genes that are either known as developmental and pluripotency or used in other species as markers, were selected for embryo viability (14, 18, 24), while they are involved in early embryogenesis (*POU5F1, CDH1, MYC* and *DPPA5*). The samples were applied for RNA isolation, complementary DNA (cDNA) synthesis and quantitative reverse transcriptase-polymerase chain reaction (qRT-PCR) analysis. The extraction of total RNA from samples, cDNA synthesis, and qRT-PCR analysis were carried out according to the protocol explained in the previous studies (25, 26).

In summary, sample was pipetted into the eppendorf tube containing 1.5 µl of lysis buffers. cDNA was synthesized by adding 2 µl poly-N and 5 µl nuclease free water to each 2 µl embryo sample. The samples were inserted in a BioRad thermocycler for 5 minutes at 75°C for the performance of reaction. By performing reverse transcription (RT), the tubes were placed on ice. 200 u RT enzyme (1 µl), 5× RT buffer (5 µl), 10 mM dNTP (3 µl), and 10 u RNase inhibitor (0.25 µl, all from Sigma) were added to the reaction. RT reaction was performed at 25°C for 10 minutes, 37°C for 15 minutes, 42°C for 45 minutes and 72°C for 10 minutes. Then, the samples were kept at 4°C overnight.

Primer sequences were used for qRT-PCR, to study the expression levels of *POU5F (OCT4), CDH1, MYC* and *DPPA5* using Rotor Gene Q instrument (Qiagen, USA) (Table 1). According to the DNA Master SYBR Green I mix manuals (Roche Applied Sciences, USA), we performed RT-PCR reactions in a total volume of 13 µl using 1 µM of each specific primer for the individual genes and 1 µM synthesized cDNA. The reaction conditions were 5 second at 95°C and 49 extension cycles of incubating 3 minutes at 95°C for denaturation, 15 seconds at 60°C, 10 seconds at 72°C for amplification. Melting curve analysis was applied to approve the single gene- specific peak of all amplification reactions. *Beta2M* was applied as an endogenous reference gene for *POU5F1, CDH1, MYC* and *DPPA5* to normalize the qRT-PCR. The authors carried out three replications and normalized fold-changes at each sample to that of endogenous internal mRNA levels (25, 26). 

**Table 1:** Details of primers used in quantitative real time polymerase chain reaction


Gene name	Primer sequence (5ˊ-3ˊ) (50–30 orientation)	Gene Bank Accession no. dents

*MYC*	F: AGC GAC TCT GAG GAG GAA C	H.CC1.1C
	R: CTG CGT AGT TGT GCT GAT G	H.CC1.2C
*POU5F1 (Oct4)*	F: CGC CGT ATG AGT TCT GTG	H.SC1.1E
	R: GGT GAT CCT CTT CTG CTT C	H.SC1.2E
*DPPA5*	F: AGT CTT CAG ACC TCA CCG AG	H.SC2.1F
	R: ACT GGT TCA CTT CAT CCA AGG	H.SC2.2F
*CDH1*	F: GCT CTT CCA GGA ACC TCT G	H.SC2.1G
	R: GGA TCT TGG CTG AGG ATG G	H.SC2.2G
*Beta2M*	F: ATG CCT GCC GTG TGA AC	H.IC1.1C
	R: ATC TTC AAA CCT CCA TGA TG	H.IC1.2C


## Statistical analysis

All statistical analyses were performed using the Statistical Package for Social Sciences software, version 22 (SPSS, USA). The means of embryo quality score, cleavage, and fertilization were measured using the nonparametric analysis test (Mann-Whitney U-test). REST 2009 software (Qiagen) was used to analyze relative levels of gene expression for various genes of embryos from two groups. The data are expressed as percentage means ± SEM. P<0.05 was considered as statistically significant. 

## Results

### The maturation of oocytes after 24-30 hours culture

Germinal vesicle oocytes were collected from the couples who were treated (out of 45 couples). 3-6 germinal vesicle oocytes were collected from each patient on average (Table 2). The data of 19 couples undergoing ICSI-PGD cycles were used (Table 3).

In a total number of 217 germinal vesicle oocytes, 144 (63.18%) reached to the stage of metaphase MII and 19 (8.83%) reached and arrested in the stage of metaphase MI. In the germinal vesicle, 54 (27.09%) oocytes were arrested. 

**Table 2:** The maturation of oocytes after 24-30 hours culture


The total number of germinal vesicle oocytes	Nuclear phase oocytes cultured *in vitro* after 24-30 hours		
	GV arrest n (%)	MII n (%)	MII n (%)

217	54 (27.09)	19 (8.83)	144 (63.18)


**Table 3:** Demographic characteristics of all evaluated intracytoplasmic sperm injection (ICSI) cycles


Characteristic	Group	
	*In vitro* oocytes n or mean ± SEM	*In vitro* oocytes n or mean ± SEM

Number of cycles	45	19
Female age (Y)	30.2 ± 1.9	29.5 ± 1.8
No. of retrieved oocytes	217	152
No. of injected oocytes	144	141
Fertilization	54.71 ± 2.08	83.84 ± 2.80
Cleavage	40.33 ± 3.82	79.61 ± 2.21
Embryo quality score	2.54 ± 0.67	2.56 ± 0.31
Four-cell embryos	72.12 ± 1.63	95.34 ± 2.65
Eight-cell embryo	70.69 ± 1.39	88.64 ± 1.72


### Fertilization and embryo development after intracytoplasmic sperm injection

There was no significant variation between two groups, regarding the injected oocytes number. The embryo quality score, cleavage and fertilization rate of the two groups are described in Table 3. In both groups, good quality (A-B) of embryos was used. Those embryos with normal morphology were chosen for molecular investigations. The rate of fertilization was significantly different between *in vitro* group (54.71% ± 2.08) and *in vivo* group (83.84 % ± 2.80, P=0.003). The cleavage rate was different among these groups (40.33 % ± 3.82 vs. 79.61 % ± 221, P=0.0001). Embryo quality score was decreased *in vitro* compared to *in vivo*. However, no statistically significant difference was observed in embryo quality score between *in vitro* (2.54 ± 0.67) and *in vivo* (2.56 ± 0.3, P=0.733). Rates of 4-cell embryo formation on day 2 of embryogenesis was significantly different between *in vitro* (72.12% ± 1.63) and *in vivo* (95.34 % ± 2.65, P=0.005). Besides, rates of 8-cell embryo formation on day 3 of embryogenesis was significantly different between *in vitro* (70.69% ± 1.39) and *in vivo* (88.64 % ± 1.72, P=0.038). 

### Quantitative reverse transcriptase-polymerase chain reaction analysis 

**Fig.1:**
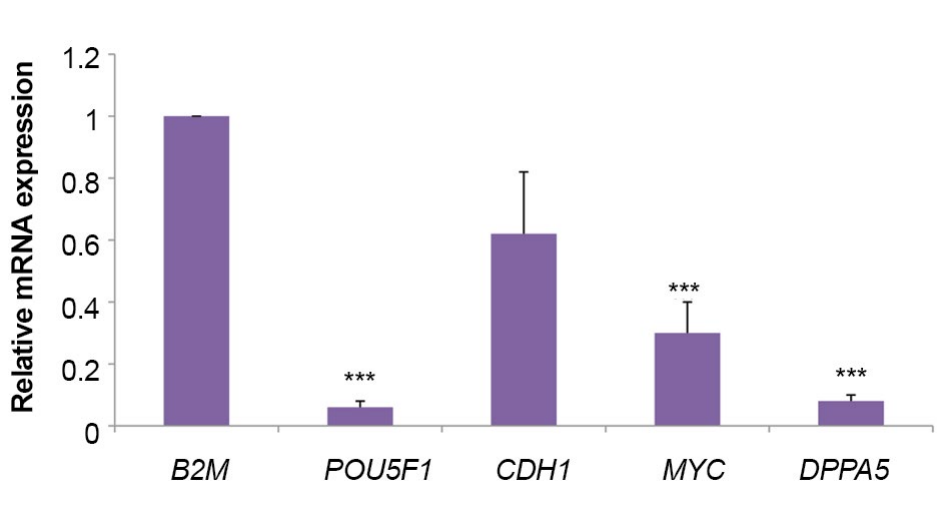
EGA related genes transcript relative quantification. Relative expression of mRNA of *POU5F1, CDH1, MYC* and *DPPA5* in 8-cell stage of human embryos *in vitro* group and *in vivo* group showed that there was a significant statistical difference (relative expression of mRNA of *POU5F1, MYC* and *DPPA5*) between the two groups. The mRNA levels of the genes were analyzed with quantitative real time-PCR. The mRNA level of each sample was normalized to those of Beta2m mRNA levels. Data are presented as mean ± SEM. ***; P<0.001.

PCR was performed in order to determine the quantitative mRNA expression profile of the implicated EGA-related genes (*POU5F1, CDH1, MYC* and *DPPA5*) in embryos originated from intracytoplasmic sperm injection. By comparing relative transcription level of *POU5F1, MYC* and *DPPA5* in embryos derived from ICSI, a significant difference was identified in the levels of pluripotency and developmental genes among *in vitro* and *in vivo* groups (all data were P=0.0001). *CDH1* gene expression was reduced *in vitro* compared to *in vivo*, while no statistically significant difference was observed between these two groups (P=0.341, Fig .1).

## Discussion

In this research, we firstly aimed to evaluate the maturation rate in IVM-GV oocytes. We used immature oocytes at the GV stages which could be obtained from patients who underwent RIF treatment (*in vitro* group). GV oocyte culture for 24-30 hours provides more embryos for patients with infertility. Routinely, our criteria for selected GV oocytes were the presence of a prominent nucleus in a homogenous cytoplasm with any type of defect in the oocyte’s overall appearance. 

In this manner, we evaluated fertilization and cleavage rate in IVM-GV oocytes with *in vivo* matured oocytes collected in couples who underwent ICSI-PGD cycles. After embryo transfer, the normal biopsied embryos excesses were donated for scientific research (*in vivo* group). We cultured GV oocytes for 24-30 hours based on IVM studies (12). The maturation rate of GV was determined to be 63.18% in our study, which is almost in agreement with other studies (27, 28). 

However, other studies reported about 35-78% IVM rate for GV oocytes collected in stimulated cycles (29, 30, 32-36). In this study, fertilization rates in GV-matured vs. *in vivo* MII oocytes were significantly different, and this is in agreement with the report of Kim et al. (27) demonstrating that rates of fertilization are significantly different in the IVM and *in vivo* groups. In the present work, the percentage means of cleaving embryos which could reach to the 4-cell stage was significantly different *in vitro*, compared to the *in vivo*. It was reported that embryos had decreased in quality, and 4-cell embryos were significantly lower in the IVM group on the second day (28).

In another study, the rate of IVM cleaved embryos approaching to the four-cell step after 40 hours was 84.5% and it was not considerably different from *in vivo* group (27). Some studies reported that oocyte maturation and fertilization rates have been 63 and 62% in PCO and PCOS cases (34 IVM cycles) respectively (29), while these rates were 74.3 and 72.6% for maturation and fertilization in 63 regular cycles (30).

There are variations in the rate of oocyte maturation, fertilization and cleavage of IVM oocytes in different studies. Several factors affect maturation of oocytes. Therefore, many differences are expected between these results. However, it would be reasonable to indicate that a crucial factor is related to the culture of immature oocytes. Thus, nuclear and cytoplasmic maturation must be considered together for immature oocytes maturation (3). So, we can conclude that the IVM process could be effective in the oocytes maturation, in any growth phase intervention, and it would affect the next embryo development. It should be noted that morphological criteria and morphometric measures of oocytes in GV stage, as nuclear and cytoplasmic competence predictor, were not reliable (31).

To assess and predict an ART program success, oocyte morphological analysis, as an oocyte quality marker, is applied using phase contrast microscopy (32). Comparison of the maturation and fertilization rates in these two groups showed that this simple and practical criterion is not trustable for the GV oocytes selection and more attention should be paid to the conditions of GV oocytes culture. Culture condition was greatly affected by oocyte maturation *in vitro*. Moreover, the percentage of oocytes developing blastocyst stage is an appropriate indicator of suitable conditions for the next oocyte development stages as well as the next embryo. Nevertheless, evidences show that morphological assessment could not always be a benchmark determinant for the fertilized oocyte and competence development (33). Results of this study indicated that, in addition to certainty of the IVM culture medium maturity and appropriate morphology of the oocytes, other factors in the medium of IVM culture and oocytes can be effective in the next steps of embryo development. 

Our second purpose was to compare gene expressions related to EGA in human embryos generated from immature and mature oocytes (matured *in vivo* and in vitro, prior to exposure to sperm) recovered from women undergoing gonadotrophin treatment for ART. Pluripotent maintenance is considered to be among the most important processes that can be altered during embryo culture. Pluripotency is largely controlled by three genes: *Oct4 (Pou5f1), Nanog* and *Sox2* (24). We assessed the EGA- related genes, including *POU5F1, CDH1, MYC (c-MYC)* and *DPPA5* (9), in two groups. This is the first research exploring the function of EGA-related genes pattern in ART (embryo derived from oocytes matured *in vitro*).

In the previous investigations, embryonic development was inhibited before reaching to the morula stage due to prohibiting transcription with a-amanitin (34). Development, under suboptimal *in vitro* conditions, will not go beyond the stage where embryonic genome is activated (35). Therefore, we proposed that ART could profoundly impress the EGA pattern in the primary stage of embryo development.

Previous studies showed while only about 50% of the zygotes are able to progress into blastocyst stage, about 80% of *in vitro*-matured and *in vitro*-fertilized bovine oocytes may reach high cleavage rates (36-39). This highlights culture periods and conditions importance for the production, viability and blastocysts quality. Several bovine studies showed that in addition to the role of the oocyte quality, the period of post-fertilization culture conditions could have great influences on the gene expression patterns responsible for the development of embryo (16).

In this study, related genes to EGA process were evaluated. *POU5F1* and *DPPA5* are known to not only be involved in the maintenance of embryonic stem (ES) cell pluripotency, but also play a key role in the embryos development. It was reported that POU5F1 null homozygous embryos are arrested by the implantation time (40). 

MYC is a regulator gene coding a transcription factor. The multifunctional protein encoded by this gene is a nuclear phosphoprotein, which has an important role in progression of cell cycle and cellular transformation (41). *DPPA5* is another gene playing role in the maintenance of ES cell pluripotency. In this study, the *POU5F1, MYC* and *DPPA5* gene expressions showed a significant decrease *in vitro* group in comparison with *in vivo* group embryos, despite the normal morphology for these embryos). No statistically significant difference was observed for embryo quality score *in vitro* group versus *in vivo*. *CDH1* gene expression was reduced *in vitro* compared to *in vivo*, but no statistically significant difference was observed between these two groups. This study showed that IVM has a negative influence on the level of the pluripotency as well as developmental and EGA-related gene expressions, in the primary stages of human embryos development. This decline in a critical developmental gene expression can negatively affect the subsequent development of embryos. Various factors can be effective in gene expression and IVM process, decreasing the introduced genes expression through affecting important factors. Thus, perhaps the reason for the low rate of pregnancy and implantation in IVM process is the lack of critical developmental-related genes during the IVM process, considering that typical pregnancy rates with IVM were determined to be 30-35% per retrieval with 10-15% implantation rates (3). 

Given the importance of these genes in embryonic development, the influence of IVM process on expression of such genes could not be ignored. RNAs and proteins are accumulated in the oocyte cytoplasm during oocytes growth phase, supporting the early phase of embryonic development before the activation of embryonic genome (42, 43). During the growth of oocyte and folliculogenesis, especially at the end of oocyte growth phase, the embryonic genome could find an opportunity to activate, due to the accumulation and subsequently degradation of many maternal mRNA species (44). During zygotic genome activation (ZGA), necessary amount of maternal factors may play a significant role (45). 

For synthesis and accumulation of maternal factors, the process of oocytes maturation should be completed including nuclear and cytoplasmic maturations. If these agents activity change, the EGA process will be affected. The quality of oocyte culture medium is one of the crucial factors that can affect oocyte maturation and synthesis of maternal factors during IVM processing (46). Therefore, molecular markers play a key role in evaluating the technical quality of IVM through embryonic development stages and our gene transcription knowledge. However, further studies would be necessary to evaluate the exact cause of reduced level of the introduced gene expressions and the IVM process improvement, to minimize the negative effects of IVM and enhance the embryo growth.

## Conclusion

It is widely accepted that maternal instructions greatly affect the embryonic development primary stages, which are fully loaded into the oocyte in the form of mRNA and proteins. Eventually, this maternal program controls the ZGA. This study showed that IVM process has a negative influence on the fertilization and cleavage rate as well as the pluripotency and developmental gene expression levels (*POU5F1, MYC* and *DPPA5*) in human preimplantation embryos. It can be deduced that normal embryo morphology cannot be a suitable scale for successful development of embryo during preimplantation stages. Therefore, further studies would be necessary to examine the molecular level and improve culture media for IVM. 
